# Supplementary material for: Gut Microbiota Enterotype as a Predictor of Sarcopenia in the Japanese Elderly Population
Source: Nutrients. 2025 Oct 16;17(20):3250. doi: 10.3390/nu17203250 (PMC12567451; doi:10.3390/nu17203250)
Supplement: Supplementary file 1 [file nutrients-17-03250-s001.zip › Supplementary_data.pdf]

Gut Microbiota Enterotype as a Predictor of Sarcopenia in the Japanese Elderly Population

Sayaka Hotta[1], Michiko Matsunaga[2], Akimitsu Miyake[3], Aya K. Takeda[4], Satoshi Watanabe[4], Naoki Hosen[5], Keisuke Hagihara[6]

1 Department of Respiratory Medicine and Clinical Immunology, Graduate School of Medicine, Osaka University, Suita, Osaka 565-0871, Japan
2 Graduate School of Education, Kyoto University, Kyoto, Japan
3 Tohoku University School of Medicine, Sendai, 980-8575, Japan
4 Cykinso, Inc., Tokyo 151-0053, Japan
5 Department of Hematology and Oncology, Graduate School of Medicine, Osaka University, Suita, Osaka, Japan
6 Research Institute for Microbial Diseases (RIMD), Osaka University, Suita, Japan

Corresponding author: Keisuke Hagihara
Research Institute for Microbial Diseases (RIMD), Osaka University, Suita, Japan
3-1 Yamadaoka, Suita, Osaka 565-0871, Japan
Tel: +81-06-6879-4261, Fax: +81-06-6879-8376
E-mail: k.hagihara@kanpou.med.osaka-u.ac.jp-

Supplementary data

Table S1. Characteristics of the participants in Study 2. Abbreviations: J-CHS, Japanese version of Cardiovascular Health Study criteria; KCL, Kihon Checklist; BMI, Body Mass Index; SMI, Skeletal Muscle Mass Index.

Characteristics	n=145
Age (y)	76.4±5.3
Sex, male, n (%)	37 (25.7)
J-CHS, n (%)	
Robust	86 (59.3)
Pre-Frailty	55 (37.9)
Frailty	4(2.8)
Sarcopenia, n (%)	
Robust	125 (86.2)
Sarcopenia	20 (13.8)
Severe Sarcopenia	0 (0.0)
KCL, n (%)	
Robust	67 (46.2)
Pre-Frailty	67 (46.2)
Frailty	11 (7.6)
Physical activity	
Gait speed, m/s	1.33±0.24
Two-step value	1.27±0.16
Grip strength, kg	25.4±6.88
Body composition	
BMI, kg/m^2	22.9±2.81
SMI, kg/m^2	6.16±0.85
Phase angle, °	4.46±0.58

Table S2. Longitudinal changes in enterotype composition observed in Study 2. Table showing the transition of enterotype classification in individual participants before and after the observation period.

ET before	ET after	n (%)
B1	B1	28 (70.0)
B1	B2	5 (12.5)
B1	R	4 (10.0)
B1	P	3 (7.5)
B2	B2	24 (70.5)
B2	B1	7 (20.6)
B2	P	2 (5.9)
B2	R	1 (2.9)
P	P	20 (80.0)
P	R	5 (20.0)
R	R	36 (78.2)
R	B1	5 (10.9)
R	P	3 (6.5)
R	B2	2 (4.3)

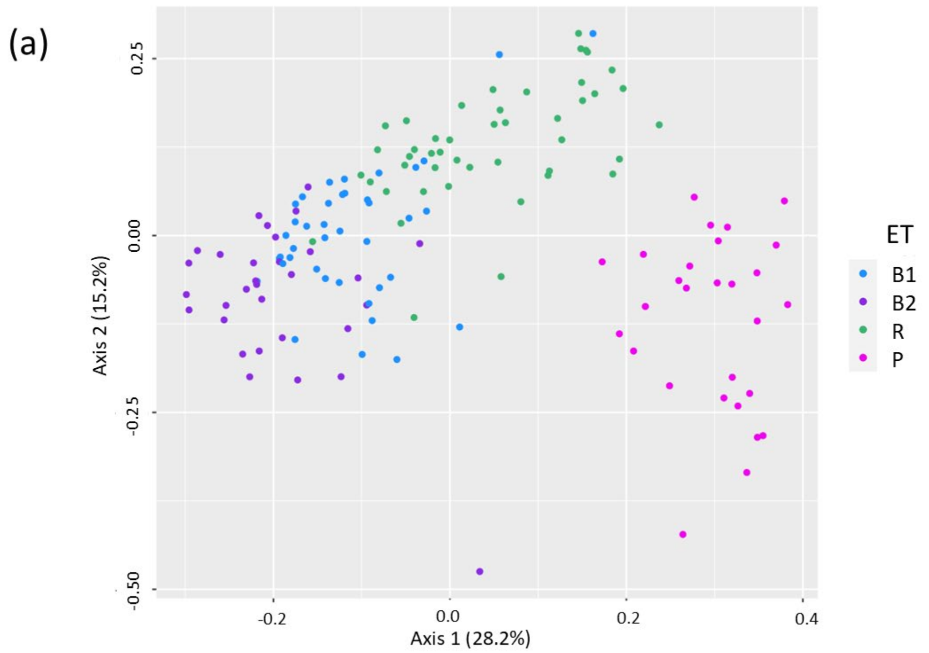

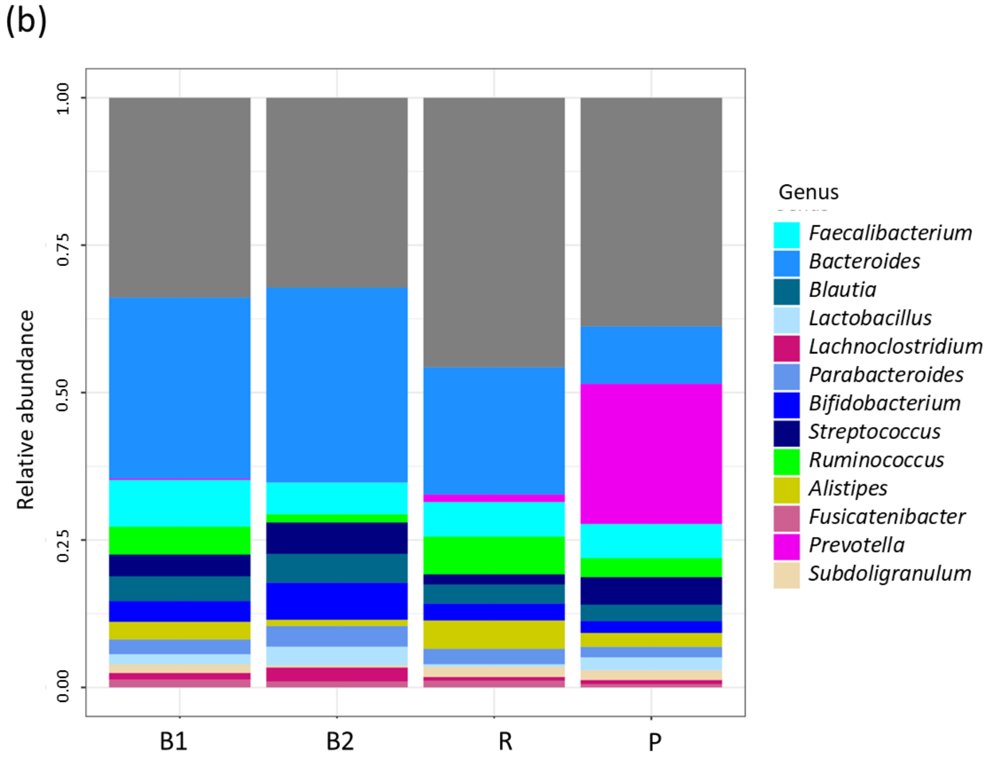

Figure S1. Beta-diversity and taxonomic composition by enterotype in Study 2. (a) Beta-diversity was assessed using principal coordinates analysis based on the Dirichlet multinomial

mixtures model, colored by enterotype. (b) Taxonomic composition showing the relative abundance of dominant genera by enterotype.

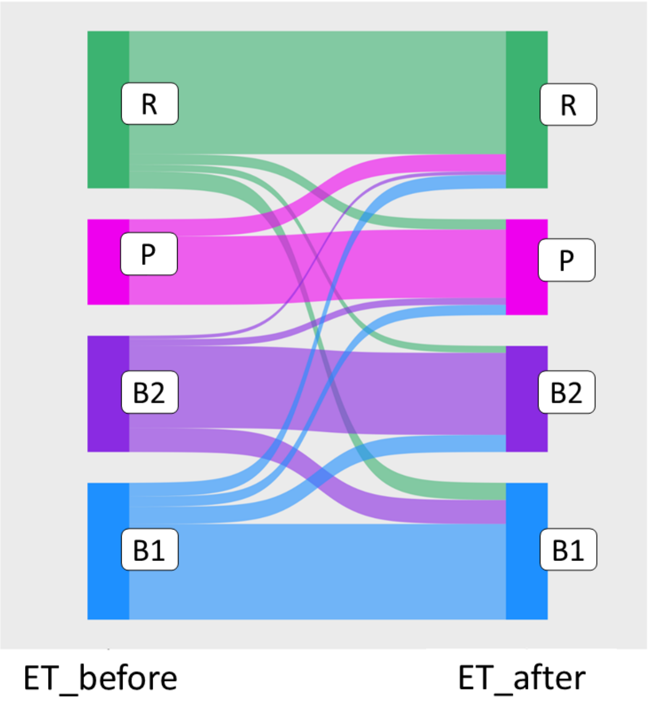

Figure S2. Figure showing longitudinal changes in enterotype composition observed. Bar plots showing the distribution of enterotypes before and after the observation period. Abbreviation: ET, enterotype.
