# Supplementary figures and images for: Gut Microbiota Enterotype as a Predictor of Sarcopenia in the Japanese Elderly Population
Source: Nutrients. 2025 Oct 16;17(20):3250. doi: 10.3390/nu17203250 (PMC12567451; doi:10.3390/nu17203250)

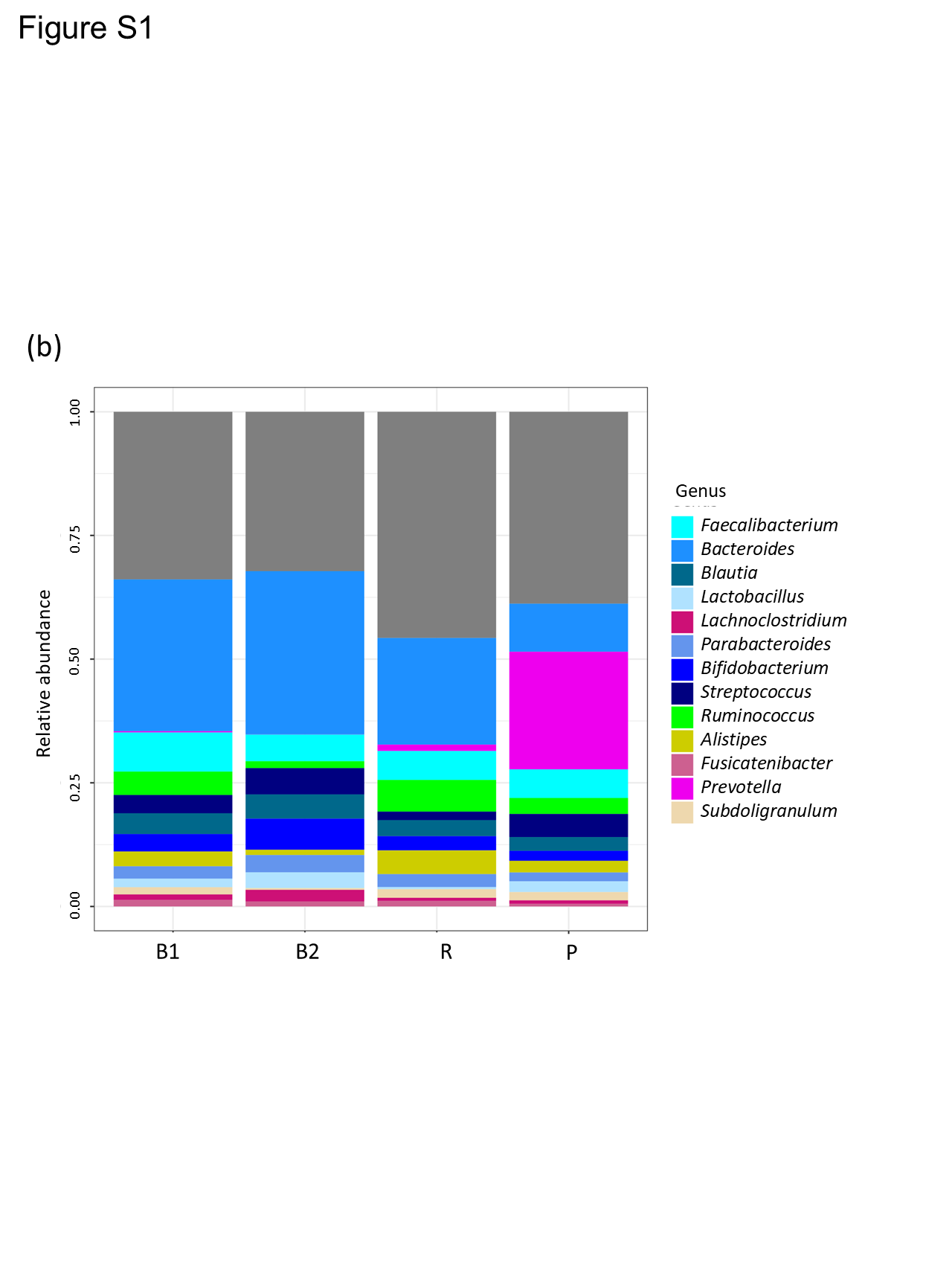

Supplement: Supplementary file 1 [file nutrients-17-03250-s001.zip › Fi.S1b.TIF]

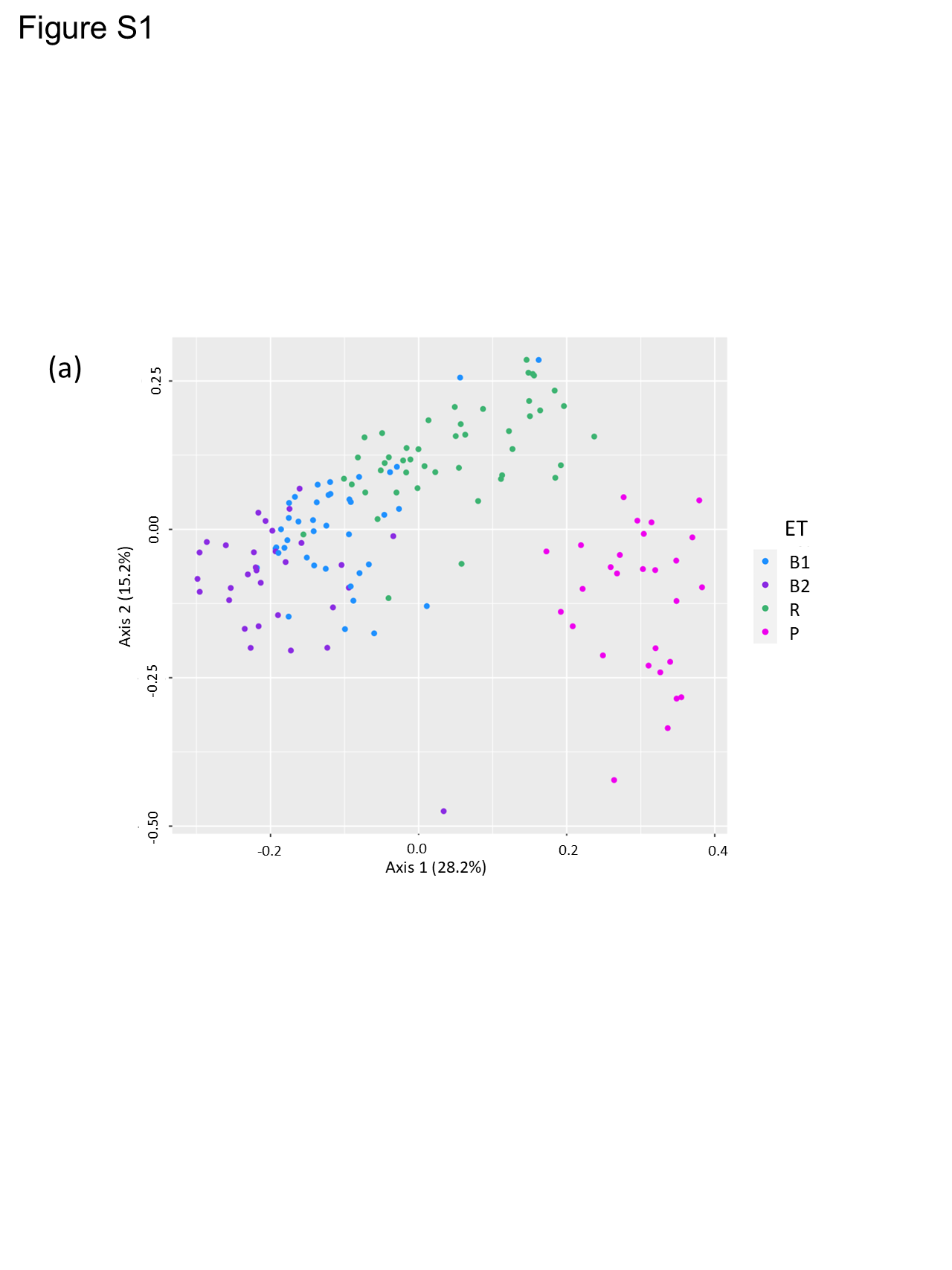

Supplement: Supplementary file 1 [file nutrients-17-03250-s001.zip › Fig.S1a.TIF]

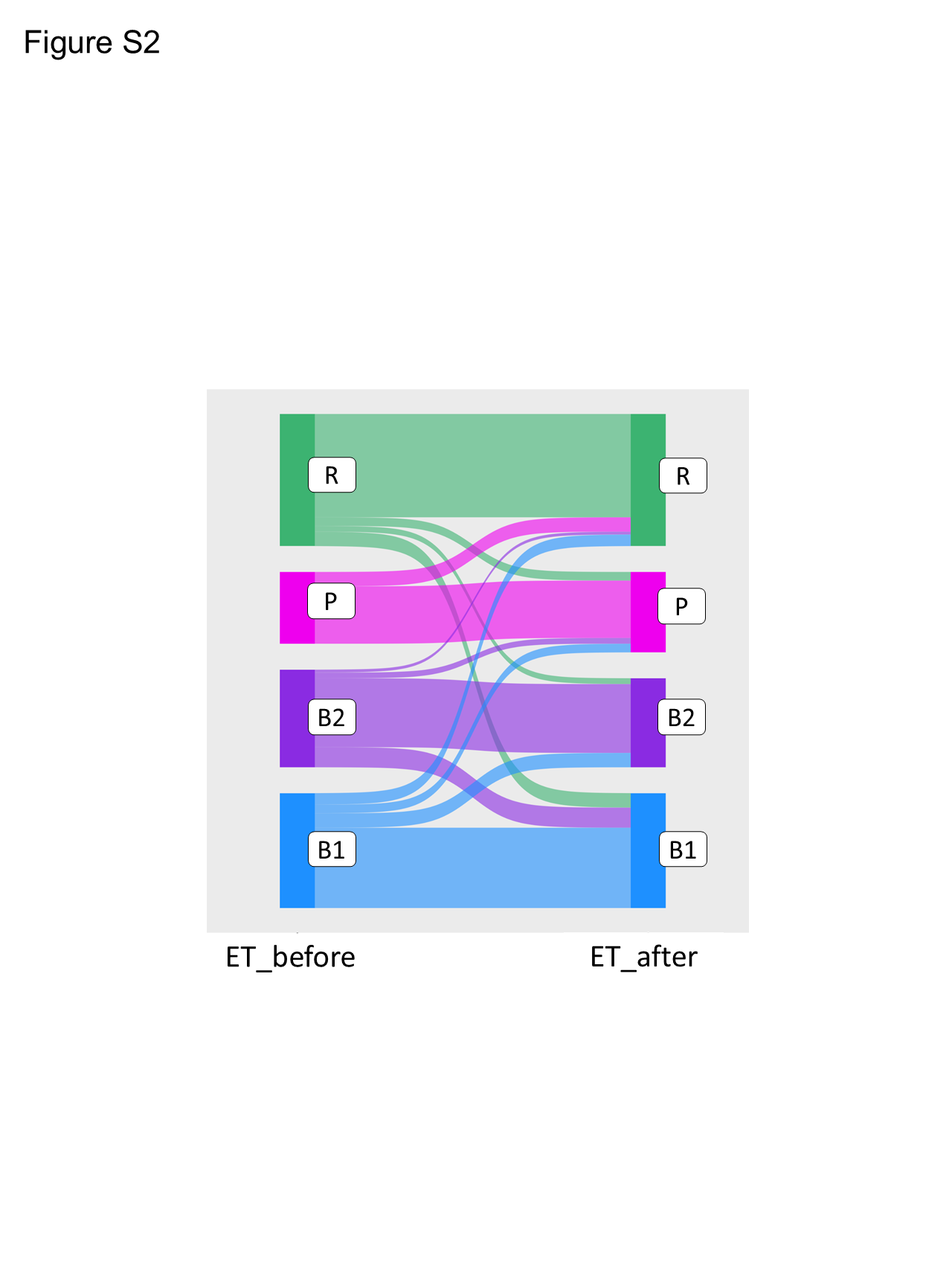

Supplement: Supplementary file 1 [file nutrients-17-03250-s001.zip › Fig.S2.TIF]
